# A nanoluciferase-encoded bacteriophage illuminates viral infection dynamics of *Pseudomonas aeruginosa* cells

**DOI:** 10.1093/ismeco/ycae105

**Published:** 2024-08-22

**Authors:** Sophia Zborowsky, Quentin Balacheff, Ioanna Theodorou, Rokhaya Kane, Raphaëlle Delattre, Joshua S Weitz, Régis Tournebize, Laurent Debarbieux

**Affiliations:** Institut Pasteur, Université Paris Cité, CNRS UMR6047, Bacteriophage Bacterium Host, 75015 Paris, France; Institut Pasteur, Université Paris Cité, CNRS UMR6047, Bacteriophage Bacterium Host, 75015 Paris, France; Institut Pasteur, UTechS Photonic Bioimaging, C2RT, 75015 Paris, France; Institut Pasteur, Université Paris Cité, CNRS UMR6047, Bacteriophage Bacterium Host, 75015 Paris, France; Institut Pasteur, Université Paris Cité, CNRS UMR6047, Bacteriophage Bacterium Host, 75015 Paris, France; Université Paris Cité, INSERM U1137, IAME, F-75006 Paris, France; Department of Biology, University of Maryland, College Park, MD 20742, United States; Institut de Biologie, École Normale Supérieure, 75005 Paris, France; Institut Pasteur, UTechS Photonic Bioimaging, C2RT, 75015 Paris, France; Centre d'Immunologie et des Maladies Infectieuses (CIMI), Sorbonne Université, INSERM U1135, 75013 Paris, France; Institut Pasteur, Université Paris Cité, CNRS UMR6047, Bacteriophage Bacterium Host, 75015 Paris, France

**Keywords:** bacterial detection, luminescence, microscopy, infection kinetics, phage therapy, host range, one step growth

## Abstract

Bacteriophages (phages) are increasingly considered for both treatment and early detection of bacterial pathogens given their specificity and rapid infection kinetics. Here, we exploit an engineered phage expressing nanoluciferase to detect signals associated with *Pseudomonas aeruginosa* lysis spanning single cells to populations. Using several *P. aeruginosa* strains we found that the latent period, burst size, fraction of infected cells, and efficiency of plating inferred from fluorescent light intensity signals were consistent with inferences from conventional population assays. Notably, imaging-based traits were obtained in minutes to hours in contrast to the use of overnight plaques, which opens the possibility to study infection dynamics in spatial and/or temporal contexts where plaque development is infeasible. These findings support the use of engineered phages to study infection kinetics of virus-cell interactions in complex environments and potentially accelerate the determination of viral host range in therapeutically relevant contexts.

## Introduction

Bacteriophages (phages) are viruses that exclusively infect bacteria, leading to rapid self-amplification at population scales and depletion of the targeted bacterial population. This lysis signal, as measured via the population-scale increase of phage or the population-scale decrease of bacteria, takes many infection and reinfection cycles. Detection of the collapse of a targeted bacterial population by phages (e.g. via plaque formation or bacterial culture lysis) has been used to identify bacterial pathogens in various contexts spanning food production, environmental characterization, and health care [1, 2].

Alternatively, different reporter genes have been inserted into phage genomes to detect a fluorescent or luminescent signal or an enzymatic activity during infection. These signals are usually detected more quickly than large-scale decreases in bacterial abundances [1–3]. Recombinant phages encoding a luciferase have been shown to be the most sensitive reporters in detecting various bacterial species such as *Bacillus, Enteroccocus, Escherichia, Klebsiella, Listeria, Salmonella, Yersinia* as well as *Mycobacteria* [3–8]. Notably, we are unaware of prior reports demonstrating engineered phages encoding a luciferase reporter activity for investigation of phage interactions with *Pseudomonas aeruginosa*, even as this pathogen is the target of ongoing clinical and experimental phage therapy studies [9, 10].

The luciferase NanoLuc (Nluc) is a 19 kDa protein that produces a strong luminescent signal when using an imidazopyrazinone substrate [11]. The gene encoding Nluc has already been cloned into phages used to detect *Streptococcus* [12], *Escherichia coli* [13], *Klebsiella pneumonia* [6], and *Enterococcus* spp [12]. Here, we report on the use of the engineered phage LUZ19 encoding Nluc (see Methods) that we named nanoLUZ19. LUZ19 is a podovirus isolated on strain PAO1 of *P. aeruginosa*, an opportunistic pathogen ranked by the World Health Organization as one of the most worrisome threats to human health [14, 15]. We studied phage infection using nanoLUZ19 on both population and single cell scales in four *P. aeruginosa* strains. These investigations advance efforts to utilize bioluminescent imaging as a means to connect within-cell kinetics to population-level propagation, estimate viral life history traits, and rapidly detect phage-host interactions. The imaging-based approach highlights the potential to investigate phage interactions with *P. aeruginosa* growing in complex spatial environments without relying on inferences via plaque-based assays.

**Figure 1:**
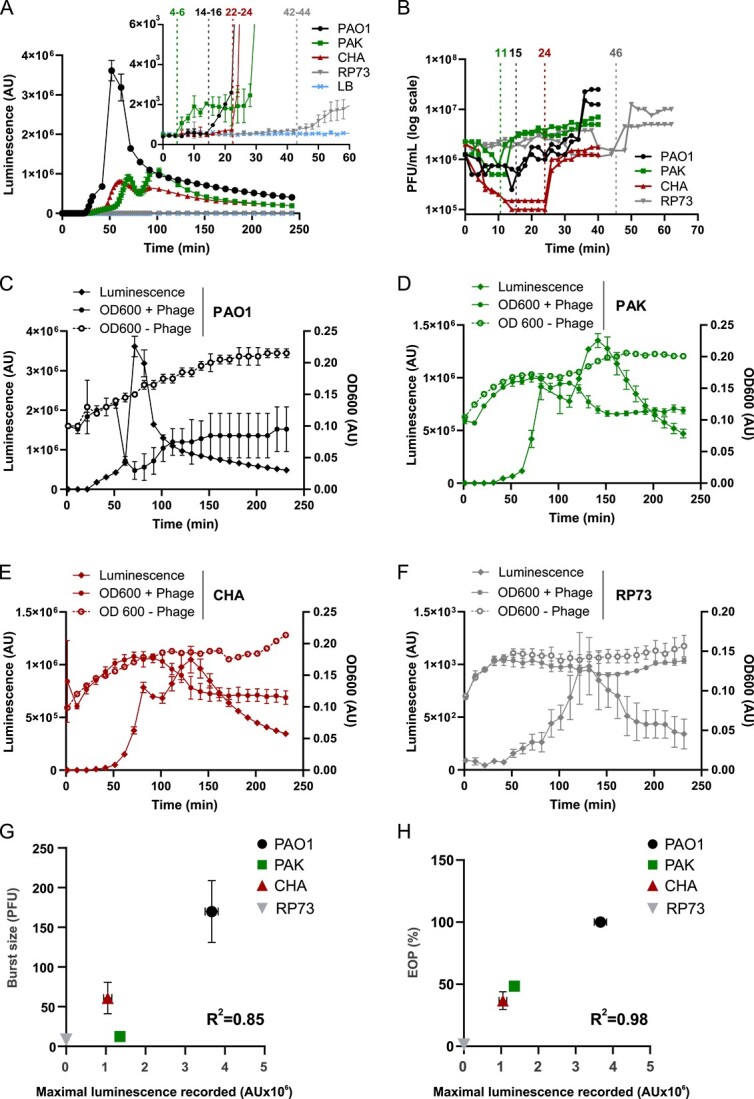
NanoLUZ19v phage produces a bioluminescent signal that reflects infection dynamics at the population scale level. (A) Time series luminescence records using a microplate reader when strains PAO1, PAK, CHA, and RP73 are independently infected by nanoLUZ19v at phage:bacteria ratio of 1:1 in presence of 1/50 dilution of the substrate (upper right corner zoomed in on the first 60 min), *N* = 3. Vertical dotted lines with digits above indicate the average time range (in min) where the signal increased above background. (B) One-step growth curves of nanoLUZ19v phage on the same strains as in panel A (*n* = 2). Vertical dotted lines with digits above indicate the average latent period (in min). (C–F) Luminescence and absorbance (600 nm) records in parallel under the same conditions as (A) for nanoLuz19v infecting (C) PAO1, (D) PAK, (E) CHA, and (F) RP73. *N* = 3 for (C–F). The maximal intensity recorded by the plate reader at a ratio of phage:bacteria of 1:1 (*n* = 3) is plotted versus: (G) the burst size as derived from one-step growth curves of nanoLUZ19v phage on the four indicated strains (*n* = 2); (H) the EOP (number of plaques formed on lawn of each strain compared to PAO1 (*n* = 4)). Average and standard deviation are plotted for both *X* and *Y* variables. The specific R^2^ value is displayed on each panel.

**Figure 2:**
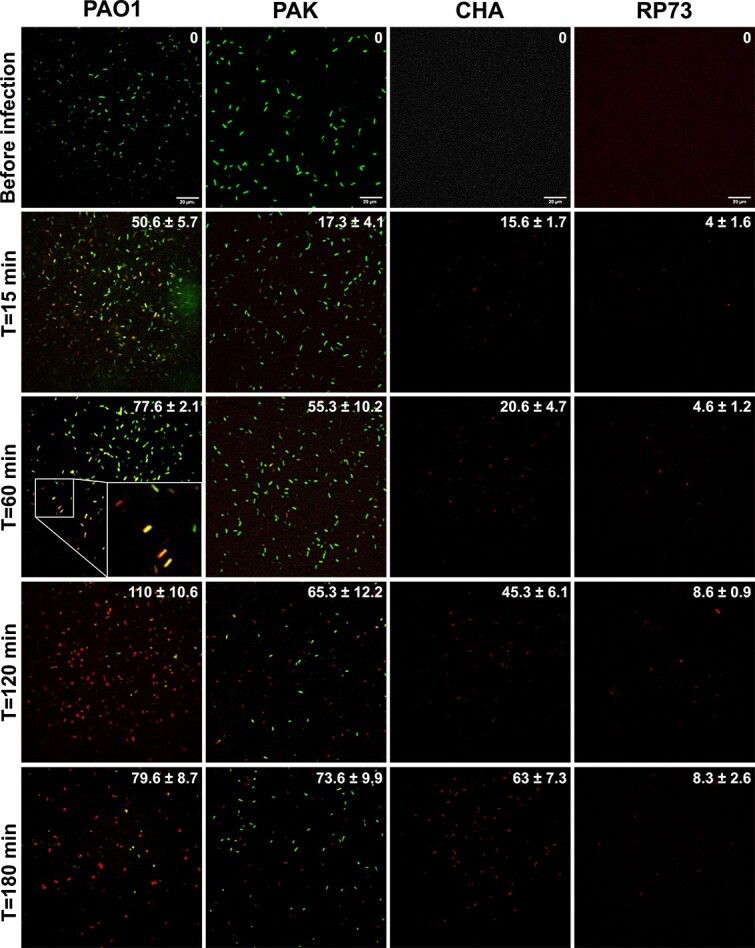
Single-cell images illuminate phage infection dynamics. Representative microscopy images of the bioluminescent signal produced by nanoLUZ19v phage-infected cells of *P. aeruginosa* strains PAO1, PAK, CHA, and RP73 (phage:bacteria ratio of 0.1:1) are shown over time (from top to bottom). Magnification 625×. Samples were taken from a liquid culture at 0, 15, 60, 120, and 180 min post phage infection, mixed with luciferase substrate and agarose and subsequently imaged using a fluorescent spinning-disk microscope. Strains PAO1 and PAK express constitutively the GFP. The 527 nm emission filter was used to detect the fluorescence originating from the GFP produced by the bacteria and is presented in the images in green. The 455 nm emission filter was used to detect luminescence produced by the phage nanoLUZ19v, presented in the images in red. For strains CHA and RP73 only luminescence could be recorded (at *t* = 0 the signal was not distinguishable from noise). Cells producing both fluorescence and bioluminescence signals appear yellow/orange depending on the relative intensity of these two signals (see zoom in the panel PAO1 *T* = 60 min). The average number of cells per field of view with standard deviation (*N* = 3) of luminescence producing cells is indicated in the top right corner of each image.

## Results and discussion

We initiated this study with phage nanoLUZ19v, a variant of the engineered phage nanoLUZ19 that was adapted to infect more efficiently strain PAK (see Methods). The luminescent signal produced by nanoLUZ19v-infected bacteria was characterized on *P. aeruginosa* strains PAO1 (efficiency of plating (EOP) of 1), PAK (EOP of 0.5), CHA (EOP of 0.4), and RP73 (EOP of 0.2). These strains were selected to compare the efficiency of signal detection and kinetics across a range of susceptibility to phage nanoLUZ19v.

Bioluminescence was measured using a microplate reader from a culture of bacteria (10^7^ cells) mixed with nanoLUZ19v (10^7^ particles) in the presence of the Nano-Glo substrate (1:50 dilution). A signal over the background was detected within 60 min for all strains however, the maximal bioluminescent intensity observed was much higher for PAO1 than for the other strains (Fig. 1A). We observed that the sharp increase in bioluminescent signal and the rapid decline in bacterial densities were concomitant (Fig. 1C–F) suggesting that the strong signal (~10^6^ AU) detected using the microplate reader originates largely from luciferase released into the medium during cell lysis. Therefore, the signal that should be emitted during the intracellular step of phage infection may be too weak to detect at the population scale by a microplate reader, e.g. because intracellular luciferase does not bind to enough levels of substrate to produce a sufficiently strong signal*.* This finding does not preclude the potential that intracellular bioluminescent activity may be detected through more sensitive optical methods (as explored below).

We then examined the relationship between life history traits inferred with luciferase-based signals and those inferred via conventional approaches. We observed that the time to detect a luciferase signal associated with bacteria lysis is consistent with the average latent period determined by one-step growth assays. We estimated an average latent period of 15 min (Fig. 1B) for PAO1, while the bioluminescent signal rose above the background on average between 14 and 16 min (Fig. 1A, Table S1); for CHA the average latent period was 24 min (Fig. 1B) and the signal rose between 22 and 24 min (Fig. 1A, Table S1); for RP73 the average latent period was 46 min (Fig. 1B) and the signal rose between 44 and 46 min (Fig. 1A, Table S1). However, for PAK the signal intensity (~10^3^ AU) increased substantially earlier (mean of 6 min) than the estimated latent period via a one-step growth curve (mean of 11 min) (Fig. 1A and B, Table S1, Fig. S1), suggesting early detection of emissions within cells. We hypothesize that substrate permeability of the bacterial envelope of strain PAK could be higher than for the three other strains. The consistency in the latent periods was observed only at a phage:bacteria ratio of 1:1 and not at lower ratios (0.1:1 and below; Fig. S2A), suggesting that the lysis of a large number of cells is required to infer the latent period from bioluminescent recordings. Conversely, when the phage input (10^7^) is constant while the bacteria input decreases, the time to detect the signal remained largely the same but the maximal intensity decreased (Fig. S2B). We have also found significant positive correlations between the intensity of the signal with burst size (R^2^ = 0.85) and with EOP (R^2^ = 0.98) (Fig. 1G and H); hence the bioluminescent activity as detected using a microplate reader can be used to rapidly infer information on life history traits.

Next, we aimed to determine if intracellular nanoLUZ19v infection events could be detected at the single cell level. Using a fluorescence microscope (see Methods), we found that bacteria producing luminescence could be visualized using the emission filter for blue light. To control which cells emit a bioluminescent signal, we used bacteria that constitutively express the fluorescent protein GFP (PAO1-GFP and PAK-GFP). We then performed time series infection experiments where the four *P. aeruginosa* strains (PAO1-GFP, PAK-GFP, CHA, and RP73) were infected in liquid medium at a phage:bacteria ratio of 1:10. At regular intervals samples from cultures were taken for imaging (Fig. 2; see Methods). Within 15 min (the minimum delay to technically perform the first observation due to the use of molten agarose) we detected luminescent cells for each condition, with the number of luminescent cells per field of view varying between strains (Fig. 2, Fig. S3A). The number of luminescent cells increased more rapidly when nanoLUZ19v infected strain PAO1-GFP compared to PAK-GFP, CHA, and RP73 strains, matching the differences observed from one-step growth infection kinetics (Table S1). For PAO1-GFP and PAK-GFP strains we calculated the ratio of cells emitting luminescence to cells expressing GFP to obtain the percentage of infected cells. At the first time point (15 min post phage addition), over 35% of PAO1-GFP cells emitted a bioluminescent signal compared to only 15% of PAK-GFP cells (Fig. S3B). The proportion of infected cells reached nearly 100% in 120 min for PAO1-GFP strain while it reached ~85% in 180 min for PAK-GFP strain (Fig. S3B).

The nanoLUZ19v reporter phage enables both (i) the detection of single cell infection events using a fluorescent microscope; and (ii) rapid estimates of infection parameters: latent period, burst size, and EOP using a plate reader that are consistent with conventional one-step growth curve estimates across four target host strains (Fig. 1G and H). Critically, this reporter-based approach circumvents the limitations of plating methods for bacteria not growing on semi-solid media or phages that do not form observable plaques. In addition, early detection of luminescent signals may help identify mechanisms underlying latent period variability, including relatively slower periods of phage maturation despite similar infection events [16].

In closing, this work demonstrates the novel use of an engineered reporter phage in *P. aeruginosa* – nanoLUZ19v that can be used to rapidly detect infections even in the absence of conventional liquid culture- or plaque-based assays. In doing so, we show that estimates of viral life history traits are consistent with conventional population-level methods and that infection can be detected rapidly at single-cell levels (often within minutes). The integration of reporter systems into phage infecting diverse pathogens—including ESKAPE pathogens like *P. aeruginosa*—using genetic tools [17] and *in vitro* synthesis techniques [18] has the potential to accelerate the assessment of phage efficacy in therapeutic contexts including multi-species biofilms and during experimental phage therapy.

## Supplementary Material

Zborowsky_Supp_revised2_f_ycae105

## Data Availability

The datasets are available at: https://doi.org/10.57745/PVPRZ9.
