# Supplementary material for: A nanoluciferase-encoded bacteriophage illuminates viral infection dynamics of Pseudomonas aeruginosa cells
Source: ISME Commun. 2024 Aug 22;4(1):ycae105. doi: 10.1093/ismeco/ycae105 (PMC11409504; doi:10.1093/ismeco/ycae105)
Supplement: Zborowsky_Supp_revised2_f_ycae105 [file zborowsky_supp_revised2_f_ycae105.docx]

**Supplementary information to the manuscript by Zborowsky *et al*. entitled**

A nanoluciferase-encoded bacteriophage illuminates infection dynamics of *Pseudomonas aeruginosa* cells.

**Methods**

**Data availability**

**Supplementary Table 1.** NanoLUZ19v infection parameters measured on different *P. aeruginosa* strains.

**Figure S1.** One step growth curves of phage NanoLUZ19v on different hosts

**Figure S2.** Intensity over time of the nanoLUZ19v phage bioluminescent signal depends on the initial input of bacteria and phage but not on the luciferase substrate concentration.

**Figure S3**. The analysis of time-lapse images of infected cells revealed phage infection dynamics.

**References**

**Methods**

*Bacterial strains and phage used in the study*

Strains PAO1 [1], PAK [2], CHA [3] and RP73 [4] were grown in LB medium at 37°C with shaking at 180rpm. Strains PAO1-GFP and PAK-GFP constitutively express the green fluorescent protein (GFP) and were provided by Darren Smith (Northumbria University, Newcastle upon Tyne UK) and Alain Filloux (Imperial College, London UK), respectively. The strain RP73 was provided by Alessandra Bragonzi (IRCSS San Raffaele Scientific Institute, Milan Italy). The phage nanoLUZ19 was originally obtained from Synthetic Genomics Vaccines Inc. (SGVI). The ownership of the phage was subsequently assigned to C3J Therapeutics, Inc., now a wholly owned subsidiary of Armata Pharmaceuticals, Inc. The Nluc gene was inserted to replace *gp49*, an ORF coding for a hypothetical protein (GeneID: 5896794) of phage LUZ19, based on data from the closely related phage phiKMV ([5] and R. Lavigne, personal communication). This phage was propagated on strain PAO1 grown in LB at 37°C.

Serial passages of phage nanoLUZ19 on strain PAK were performed in liquid LB medium by co-incubating the phage and bacteria at ratio of 1:100 for 5 h at 37°C with shaking. The lysate was filter-sterilized and titrated on both strain PAO1 and PAK. The next day a fresh culture of strain PAK was infected by this lysate at phage:bacteria ratio of 1:100. This process was repeated five times. The last lysate was spread to isolate plaques. Two plaques were randomly picked and streaked to isolate new plaques, three times. The final two plaques were amplified and titrated on both PAO1 and PAK. One of these lysates was chosen and named nanoLUZ19v. A large lysate (500 mL) was obtained from PAK-infected cells, filtered at 0.45 and then 0.2 µm (Sartopore 2, Sartorius) and then concentrated by ultrafiltration (Vivaflow 200, Sartorius) with Tris buffer exchange (10 mM Tris, 150 mM NaCl, pH 7.5). The lysate was then ultracentrifuged twice over cesium chloride gradients and dialyzed against Tris buffer. Endotoxins were removed with Endotrap HD (Lionex) high capacity resin and the residual endotoxin levels were measured using recombinant factor C. The purified phage suspension was then stored at 4°C. The genome sequence of this phage is available on Genbank (OQ652145). A single point mutation (position 28009) in *gp34* coding for the tail tubular protein B was present in 100% of the sequencing reads and represents the best candidate to explain the improved in vitro efficacy, as the two other mutations (position 10018 and 25901) were located in a gene (gp16) coding a hypothetical protein and an intergenic region, respectively.

*Bioluminescence recording during in vitro infection kinetics experiments*

Bacteria and phage were mixed with luciferase substrate (Nano-Glo Luciferase assay, Promega) at a final volume of 0.05 mL in 96 white wells, clear bottom tissue culture plate (Costar). The plate was sealed with a transparent cover and placed in an automatic plate reader (GlomaX, Promega) at 37°C. Luminescence and bacterial density were recorded every 2-10 min for 200-400 min. Prior to each measurement the plate was shaken for 10 sec (orbital shaking, 220 cycles per min, 2 mm shaking diameter). Bacterial density was measured by proxy through absorbance at 600nm. Luminescence was recorded using the 495nm Luminescence Filter Paddle with an integration time of 0.3 sec.

*One step growth assays*

One step growth assays were performed according to Chevallereau et al. [6] with the following modification. Briefly, exponentially growing bacteria were infected at a phage:bacteria ratio of 1:1000 and samples were taken in 2 min intervals from t=0 to t=40 min for PAO1, PAK and CHA strains. We extended samples withdrawing to 60 min for RP73 strain. At each time point two samples were taken, one was filtered through a 0.2 µm filter (Milex) to remove cells (assessment of the extracellular phage), and the other was mixed with CHCl3 to burst the cells (assessment of extracellular and intracellular phage). All samples were enumerated at each time point by platting serial dilutions of the phage on solid LB medium overlaid with strain PAO1. Latent periods and burst sizes were calculated using one step curves [6]. The latent period was estimated by the time point where the concentration in extracellular phage started to increase; the burst size was calculated by using the following equation:

A: Phage concentration before release without CHCl3

B: Phage concentration before release with CHCl3

C: Phage concentration at plateau after the burst without CHCl3

$$Burst Size=\frac{C-B}{A-B}$$

*Bioluminescence recording during time lapse microscopy*

Exponentially growing bacteria (2 x 10^8^ cells/mL) were mixed with phage at a phage:bacteria ratio of 1:10, and kept at 37°C, 1 mL samples were taken at 25, 60, 120 and 180 min post infection and centrifuged for 1 min at 14,000g to pellet the cells. Supernatants were discarded to remove free phage and cells were resuspended in 80 µL PBS before to be mixed with 0.75 percent ultra-pure agarose (Invitrogen) and 1:10 Nano-Glo at a final volume of 0.2 mL, of which 0.15 mL was pipetted unto a µ-slide 8 well microscope slide (Ibidi). Samples were imaged using the Spinning-disk UltraVIEW VoX (Perkin-Elmer) microscope (oil immersion, 25X magnification). Luminescence was recorded using the emission filter for “blue” light (405-475nm), at maximal sensitivity and with 4 s exposure time. The excitation laser power was set to zero. Green fluorescence was recorded with excitation/emission of 488/550nm (sensitivity 120, exposure time 0.3 s for PAK and 3 s for PAO1). Images were acquired using the Volocity software (v3.6.1beta) and later processed using ImageJ. Cells were distinguished from noise by removing the background and filtering based on size (area between 2-8 pixels^2^).

**Data availability**

The datasets generated and/or analyzed during the current study are available at:

<https://entrepot.recherche.data.gouv.fr/privateurl.xhtml?token=5c0dc7c9-597a-4bbc-85e9-84434197435e>

**Supplementary Table 1.** NanoLUZ19v infection parameters measured on different *P. aeruginosa* strains by luminescence, one step growth and plating

| Strain | Time to signal increase (min)^a^ | Latent period (min)^b^ | | Burst size (PFU)^b^ | | EOP relative to strain PAO1 (%)^c^ |
| --- | --- | --- | --- | --- | --- | --- |
|  |  | Rep 1 | Rep 2 | Rep 1 | Rep 2 |  |
| PAO1 | 14-16;14-16;14-16 | 14.0 | 16.0 | 197.6 | 142.3 | 100.0 |
| PAK | 4-6;6-8;4-6 | 12.0 | 10.0 | 13.8 | 11.0 | 48.3±2.3 |
| CHA | 22-24;22-24;22-24 | 24.0 | 24.0 | 75.0 | 47.0 | 36.7±6.2 |
| RP73 | 40-42;42-44;46-48 | 48.0 | 44.0 | 5.1 | 11.0 | 0.2±0.1 |

^a^ time at which the signal rises above the background (LB alone) for the condition of bacteria:phage ratio of 1:1. Values for three biological replicas shown.

^b^ Latent period and burst size were determined from one step growth curves (the two replicas are labelled Rep 1 and Rep 2).

^c^ EOP (efficiency of plating) was measured by spotting the phage on a lawn of the indicated bacteria and counting the plaques formed (N=4).





**Figure S1.** One step growth curves of nanoLUZ19v phage on (A) PAO1, (B) PAK, (C) CHA, (D) RP73 strains. Phage concentration with and without CHCL3 treatment of the samples (see methods) are displayed. The latent period for each replica is indicated by the dotted line and its value is written above the line (in min).





**Figure S2.** Intensity over time of the nanoLUZ19v phage bioluminescent signal depends on the initial input of bacteria and phage but not on the luciferase substrate concentration.

In the two panels, the black round filled symbol corresponds to the condition of 1x10^7^ CFU, 1x10^7^ PFU (phage:bacteria ratio of 1:1) and the substrate diluted at 1/10. In panel (A) variations of initial PFU were tested (from 1:1 to 0.0001:1). In panel (B) variations of initial CFU were tested (from 1:1 to 1:0.000001). Lowering the host input decreases intensity of the bioluminescent signal, but largely does not affect the time it takes for the signal to rise over the background, whereas initial phage input largely does not affect the intensity but does shift the time it takes to rise over the background. The Y axis displays luminescence intensity in log scale (in contrast to linear scale in Figure 1) to favor the visual comparison of the conditions tested.





**Figure S3**. The analysis of time-lapse images of infected cells revealed phage infection dynamics.

(A) From microscopy images the 455nm emission filter was used to detect luminescence produced by the phage nanoLUZ19v infecting either strain PAO1, PAK, CHA or RP73, allowing to count infected cells. (B) The 527nm emission filter was used to detect the fluorescence emitted by the strains PAO1-GFP or PAK-GFP and combined with the 455nm filter led to count infected and uninfected cells. The total number of cells was calculated by adding the number of cells detected on each channel, while accounting for cells that appeared in both channels (based on co-localization of the signals when the channels were merged). Cells were counted using Image J. N=3.

**References**

1. Stover CK, Pham XQ, Erwin AL, Mizoguchi SD, Warrener P, Hickey MJ, et al. Complete genome sequence of Pseudomonas aeruginosa PAO1, an opportunistic pathogen. *Nature* 2000; **406**: 959–964.

2. Cain Amy K., Nolan Laura M., Sullivan Geraldine J., Whitchurch Cynthia B., Filloux Alain, Parkhill Julian. Complete genome sequence of Pseudomonas aeruginosa reference strain PAK. *Microbiol Resour Announc* 2019; **8**: e00865-19.

3. Delic-Attree I, Toussaint B, Froger A, Willison JC, Vignais PM. Isolation of an IHF-deficient mutant of a Pseudomonas aeruginosa mucoid isolate and evaluation of the role of IHF in algD gene expression. *Microbiology* . 1996. Microbiology Society. , **142**: 2785–2793

4. Jeukens Julie, Boyle Brian, Bianconi Irene, Kukavica-Ibrulj Irena, Tümmler Burkhard, Bragonzi Alessandra, et al. Complete genome sequence of persistent cystic fibrosis Isolate Pseudomonas aeruginosa strain RP73. *Genome Announc* 2013; **1**: 10.1128/genomea.00568-13.

5. Lavigne R, Noben J-P, Hertveldt K, Ceyssens P-J, Briers Y, Dumont D, et al. The structural proteome of Pseudomonas aeruginosa bacteriophage ϕKMV. *Microbiology* . 2006. Microbiology Society. , **152**: 529–534

6. Chevallereau A, Blasdel BG, De Smet J, Monot M, Zimmermann M, Kogadeeva M, et al. Next-generation “-omics” approaches reveal a massive alteration of host RNA metabolism during bacteriophage infection of Pseudomonas aeruginosa. *PLOS Genet* 2016; **12**: e1006134.
